# Diagnostic Tools in Allergic Rhinitis

**DOI:** 10.3389/falgy.2021.721851

**Published:** 2021-09-23

**Authors:** Almudena Testera-Montes, Raquel Jurado, Maria Salas, Ibon Eguiluz-Gracia, Cristobalina Mayorga

**Affiliations:** ^1^Allergy Clinical Unit, Hospital Regional Universitario de Málaga, Málaga, Spain; ^2^Allergy Research Group, Instituto de Investigación Biomédica de Málaga (IBIMA), Málaga, Spain

**Keywords:** allergic rhinitis, rhinitis - diagnosis, *in vivo* test, *in vitro* test, inflammatory mediator, rhinitis phenotypes, chronic rhinitis

## Abstract

Allergic mechanisms account for most cases of chronic rhinitis. This condition is associated with significant impairment of quality of life and high indirect costs. The identification of the allergic triggers of rhinitis has been historically based on the performance of atopy test [skin prick test (SPT) and serum allergen-specific (s)IgE]. Nevertheless, these tests only denote sensitization, and atopy and allergy represent two different phenomena. It is now clear that allergic phenotypes of rhinitis can exist in both atopic (allergic rhinitis, AR) and non-atopic (local allergic rhinitis, LAR) individuals. Moreover, both allergic phenotypes can coexist in the same rhinitis patient (dual allergic rhinitis, DAR). Therefore, a diagnostic approach merely based on atopy tests is associated with a significant rate of misdiagnosis. The confirmation of the allergic etiology of rhinitis requires the performance of *in vivo* test like the nasal allergen challenge (NAC). NAC is mandatory for the diagnosis of LAR and DAR, and helps decide the best management approach in difficult cases of AR. Nevertheless, NAC is a laborious technique requiring human and technical resources. The basophil activation test (BAT) is a patient-friendly technique that has shown promising results for LAR and DAR diagnosis. In this review, the diagnostic usefulness for chronic rhinitis of SPT, NAC, olfactory tests, serum sIgE, BAT and the quantification of inflammatory mediators in nasal samples will be discussed. The accurate performance of an etiologic diagnosis of rhinitis patients will favor the prescription of specific therapies with disease-modifying potential like allergen immunotherapy.

## Introduction

Respiratory allergy is a chronic IgE-mediated type 2 inflammatory disease affecting pediatric and adult populations and impacting negatively the quality of life of the patients and the economic health system (1). Allergic rhinitis (AR) is the most frequent clinical manifestation within the respiratory allergies with an estimated prevalence of 20–40% of population. AR is commonly associated with conjunctivitis and/or asthma as illustrated by the expression of “one airway one disease” concept (2).

Patients affected by AR need a proactive and individualized clinical approach, combining etiologic precision diagnosis and personalized treatment. The etiologic diagnosis, identifying the allergen or allergens clinically relevant or causative of the allergic symptoms, is essential for the prescription of a personalized treatment with specific allergen immunotherapy (AIT).

Recently, our understanding about the physiopathology of AR has increased and new allergic endophenotypes of rhinitis have been described in both non-atopic and atopic individuals (3, 4). Together with the high rate of polysensitization among AR patients and the existence of atopic patients with non-allergic rhinitis (NAR), this fact reveals the complexity of the etiologic diagnosis of rhinitis and emphasized the importance of differentiating between allergy and atopy (5).

The scope of this article includes an updated revision of the literature on the main indication, limitation, setting applicability, safety and diagnostic accuracy of the main diagnostic *in vivo* and *in vitro* tools available for allergic phenotypes of rhinitis.

## Subsections Relevant for the Subject

### Rhinitis Phenotypes and Mechanism

Chronic rhinitis is defined by the presence of ≥2 cardinal nasal symptoms (itch, obstruction, sneezing and rhinorrhea) for ≥1 h per day and during ≥12 weeks. Although the disease is highly heterogeneous, allergic mechanisms account for most chronic rhinitis cases.

Allergic phenotypes of rhinitis are characterized by the positivity of the nasal challenge with at least one relevant allergen (5). The presence of atopy, positive skin prick test (SPT) and/or detectable serum allergen-specific IgE (sIgE), further classifies allergic patients with rhinitis into several sub-phenotypes (3). AR subjects display positive SPT results with all allergens testing positive in the nasal allergen challenge (NAC) (5). AR is also the most studied phenotype and according to ARIA (*Allergic Rhinitis and its Impact on Asthma*) guidelines can be divided according to its temporality (intermittent vs. persistent) and severity (mild vs. moderate-severe). AR currently affects >20% of the Western population and >500 million people worldwide. Conversely, local allergic rhinitis (LAR) individuals display negative SPT results to all allergens testing positive in the NAC (3). Interestingly, recent data indicates that AR and LAR can coexist in the same rhinitis patient, and this phenotype has been called dual allergic rhinitis (DAR) (4). DAR patients typically display perennial nasal symptoms with seasonal exacerbations, positive SPT to seasonal allergens (pollens) only, and positive NAC with both seasonal and perennial allergens (house dust mites or molds) (6). On the other hand, NAR is defined by the negativity of the NAC with all relevant allergens (5). The coexistence of NAR and AR in the same rhinitis patients is also a common clinical scenario and this phenotype is termed mixed rhinitis (MR) (7). MR patients frequently suffer from perennial nasal symptoms with seasonal exacerbations, are sensitized to seasonal allergens only, and display positive NAC results with the allergens they are sensitized to and negative NAC results with the rest of relevant allergens.

**Figure 1:**
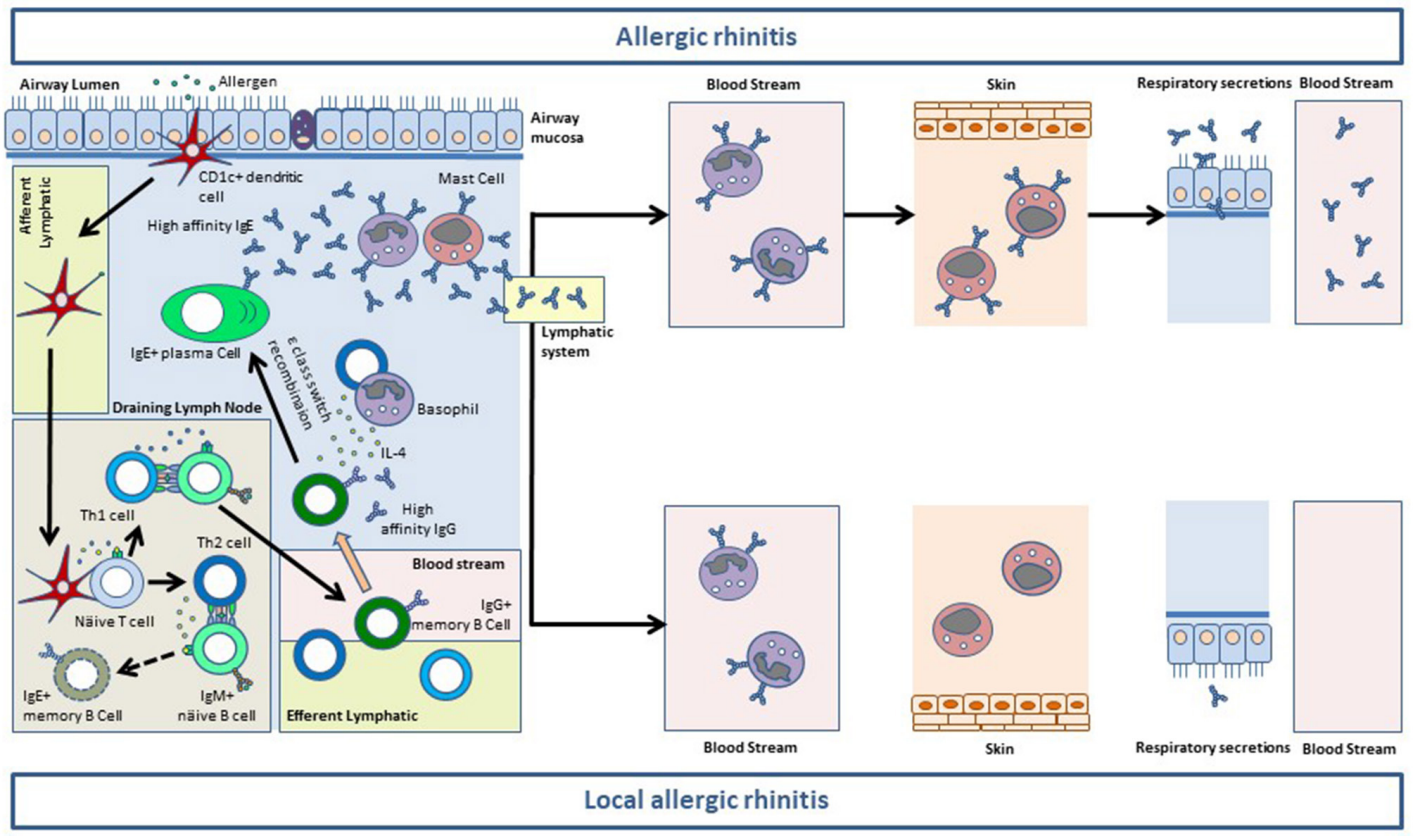
Mucosal synthesis of allergen-specific (s)IgE in the airway mucosa of allergic rhinitis (AR) and local allergic rhinitis (LAR) patients. After taking up the allergen, CD1c+ myeloid dendritic cells traffic to the germinal center of the draining lymph nodes. Here, dendritic cells activate allergen-specific naïve CD4+ T cells to generate Th1 and Th2 cells. Th1 cells stimulate allergen-specific IgM+ naïve B cells which undergo class switch recombination (CSR) to IgG and somatic hypermutation to give rise to IgG+ memory B cells (among other B and plasma cell subsets). The antibodies produced by IgG+ memory B cells display a high affinity for the allergen. Allergen-specific Th1 and IgG+ B cells exit the lymphoid system through the efferent vessels and reach the blood stream. Activated Th2 cells in the germinal centers also stimulate IgM+ näive B cells which undergo CSR to IgE. Nevertheless, IgE+ B cells cannot efficiently carry out their somatic hypermutation, and die by apoptosis before exiting the germinal centers. Conversely, allergen-specific Th2 cells reach the blood stream and extravasate to the airway mucosa, together with Th1 cells (not shown) and IgG+ memory B cells. In the lamina propia IgG+ memory B cells can undergo sequential CSR to IgE upon allergen re-encounter (not shown) and stimulation with IL-4 provided by Th2 cells and basophils. This process results in the generation of IgE+ plasma cells releasing vast amounts of high-affinity sIgE to the lamina propia of the airway mucosa. Mucosal sIgE binds to FcεRI on resident basophils and mast cells and sensitize them for activation. After saturating the FcεRI receptor system of the mucosa, sIgE reaches the blood stream to bind to FcεRI on circulating basophils. In patients with AR, sIgE saturates the receptor system of blood basophils and subsequently binds to FcεRI expressed on the mast cells residing at peripheral tissues including the skin. After saturating the FcεRI receptor system of the organism, sIgE can be found free in serum and in the respiratory secretions of AR subjects. In patients with LAR, the sIgE synthetized at the mucosal level is sufficient to saturate FcεRI on mucosal resident mast cells and basophils, and in >50% of cases is also sufficient to sensitize peripheral basophils. Nevertheless, LAR patients do not have enough sIgE to saturate FcεRI on peripheral basophils, and thus sIgE is not found on skin mast cells or serum. Most patients with LAR do not have sIgE in respiratory secretions either, although low levels are sometimes detected. This phenomenon probably corresponds to a small sIgE leakage through the epithelim, before the antibody exits the mucosa via the lymphatic system.

The immunopathology of allergic phenotypes of rhinitis is mainly mediated by the mucosal synthesis of sIgE (8). The generation of IgE^+^ memory B-cells in the secondary lymphoid tissues is impaired. Conversely allergen-specific IgG1^+^ memory B-cells complete their differentiation in the germinal centers and produce high affinity antibodies (8). IgG1^+^ memory B-cells can be recruited to the nasal mucosa where they undergo sequential class switch recombination to IgE upon the influx of IL-4 provided by basophils or Th2 cells (9). IgE synthetized at the mucosal level binds first to the high affinity receptor (FcεRI) in resident mast cells. When the receptor system of the nasal mucosa is saturated, IgE traffics to the blood stream through the lymphatic vessels where it binds to FcεRI on peripheral basophils (8). Thereafter, IgE is distributed through the organisms where it sensitizes resident mast cells, including skin mast cells. Only after the saturation of the whole receptor system of the organism, IgE can be found free in serum and other biological fluids (8). There is ample evidence showing that most serum sIgE in AR patients is synthetized at the mucosal level (10, 11). For local allergic phenotypes of rhinitis (LAR and DAR) this evidence is scarcer, although the pooled analysis of LAR patients revealed that sIgE increased in the nasal secretions during the 24 h following a positive NAC (12). Moreover, in >50% of LAR and DAR individuals, sIgE against the allergens they test negative to in SPT can be detected in the membrane of peripheral basophils (4, 13) (Figure 1).

Importantly, a positive NAC induces an eosinophilic infiltrate of the nasal mucosa in AR, LAR, DAR and MR patients regardless of the presence of atopy for the corresponding allergen (4, 14).

### Nasal Examination as 1rst Step on Diagnostic

The clinical history can guide phenotyping nasal chronic symptoms. Facial pain and headache are more common among CRS patients, whereas nasal itch and sneezing prevail in individuals with chronic rhinitis, especially those suffering from allergic phenotypes. Moreover, patients with chronic rhinitis should be subjected to a nasal examination as an essential step of the diagnostic process (5). In patients with perennial rhinitis a nasal examination is paramount to differentiate between chronic rhinitis and chronic rhinosinusitis (CRS). Subjects with smell impairment should also be subjected to a nasal examination. Smell and taste disorders are more frequent in patients with chronic rhinosinusitis (CRS) with nasal polyps (CRSwNP), but they can also be present in individuals with chronic rhinitis and CRS without nasal polyps (15). Anatomical alterations (septum deviation, turbinate hypertrophy, etc.) can also drive chronic nasal symptoms that are difficult to differentiate from inflammation-driven rhinitis on the basis of the clinical history (5).

**Figure 2:**
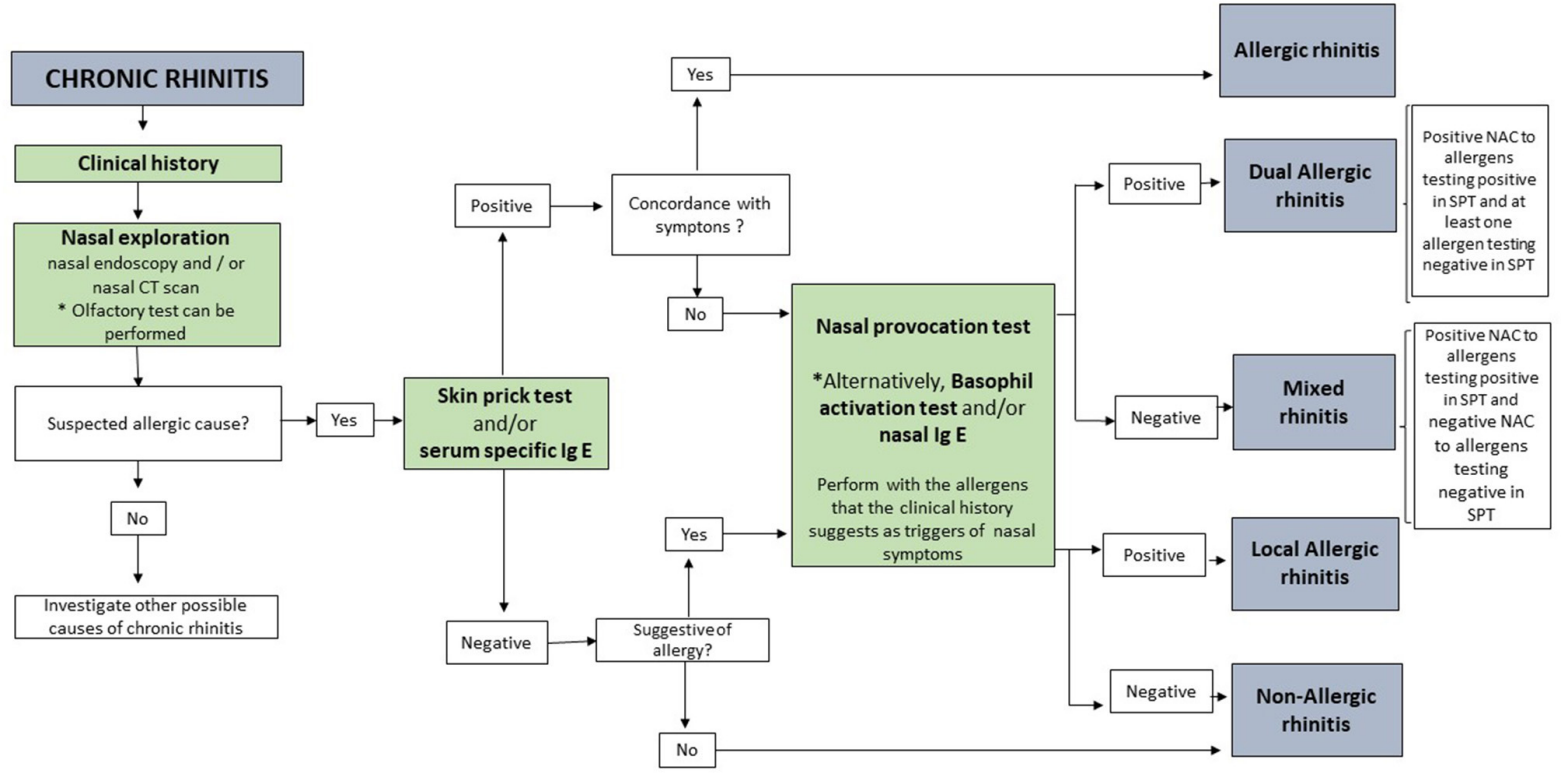
Diagnostic algorithm for chronic rhinitis.

Examination should start by a nasal inspection that can reveal severe cases of septum deviation or valve collapse. Thereafter, an anterior rhinoscopy should be performed using a manual rhinoscope and a light source. This examination assesses for the permeability of the nostrils and permits the visualization of the head of the lower turbinate. Rhinoscopy can also reveal the presence of severe nasal polyps (grade IV). If the nostrils are permeable, the rhinoscopy should be followed by a nasal endoscopy (using either rigid or flexible endoscope). The endoscope is the best way to evaluate the state of the nasal mucosa and diagnose the distinct naso-sinusal inflammatory diseases. Allergic phenotypes of rhinitis are commonly associated with pale and edematous mucosa in the anterior third of the nostril (head of the lower turbinate, axilla of the middle turbinate, etc.) together with watery secretions. Individuals with NAR or CRS without nasal polyps display less edema and the mucosa has a bumpy aspect. In patients with CRSwNP whitish polyps are commonly observed in the middle meatus (Figure 2).

### *In vivo* Tests

#### Skin Tests

Skin tests are the main *in vivo* test used to demonstrate an IgE-mediated sensitization in a patient, and represent a major diagnostic tool in Allergology. Skin tests are useful to establish the atopic state of a patient and to confirm the sensitization to allergens (16, 17). Although different skin test methods are available, SPT is the main *in vivo* diagnostic test recommended by international guidelines (18–20). The SPT modified by Pepys is currently used as a reference (21). SPT is regarded as the most sensitive and specific test to detect sensitization to allergens, thus representing the gold standard for atopy identification (22, 23). Of note, a small but consistent proportion of patients with undetectable serum sIgE, display positive SPT results, whereas the opposite scenario is rare and probably explained by methodological mistakes (4, 6). It is also a reproducible technique using standardized allergenic extracts. This test identifies allergen-sIgE bound to FcεRI molecules expressed on the membrane of skin mast cells. These cells, when activated by the allergen, release mediators that cause an itchy papule on the skin (1).

Nevertheless, a positive SPT alone does not confirm that the patient experience symptoms upon exposure to the allergen. It is necessary that the medical history is consistent with the results of skin tests to establish the diagnosis of AR (1). In patients showing discrepancies between the symptom pattern and IgE-sensitizations tests, or in patients with suspected diagnosis of local respiratory allergy (LAR or DAR phenotypes), it will be necessary to perform a NAC (Figure 2).

#### Nasal Allergen Challenge

The NAC is a medical procedure aiming to reproduce the inflammatory reaction induced by the allergen in the nasal mucosa of allergic patients in a controlled manner. From a practical point of view, the procedure involves the intranasal administration of known doses of allergen (e.g., by nasal spray, micropipette, etc.) after the exclusion of nasal hyperreactivity (as measured by a control challenge). The NAC is a safe and reproducible technique (3, 24, 25), and is considered the gold standard to identify the allergic triggers of rhinitis. Therefore, the NAC permits a confirmation diagnosis of AR, LAR, DAR and occupational AR. Additionally, the NAC is a useful tool used in research studies to investigate the mechanisms of nasal inflammation and monitor available therapies. Despite this aspects, NAC is poorly implemented due to the absence of standardized protocols, among other aspects. In this regard, the consensus documents of the European Academy of Allergy and Clinical Immunology (EAACI) (26) and the Spanish Society of Allergy and Clinical Immunology (SEAIC) (27) guideline advise evaluating NAC through subjective parameters (symptom score) and objective parameters (nasal patency). However, they differ in the symptom score used (VAS, Linder or Lebel score…), in the method for evaluating nasal patency (nasal inspiratory peak-flow, acoustic rhinometry, active anterior rhinomanometry…) and in the cut-off points of positivity (26, 27).

It is important to take into account that NAC should be performed using standardized allergenic extracts applied bilaterally by micropipette or nasal spray, both procedures being equally safe (25). Existing guidelines recommend administering only one allergen per session. However, NAC is a time-consuming procedure (26), which is solved by using the multiple NAC protocol that allows 4 allergens to be administered consecutively. This protocol was validated 10 years ago and represent a safe and reproducible technique (28).

The positivity criteria of the different guidelines are variable, although the NAC is considered positive if the patient experiences a very significant change in nasal patency and/or in symptom score or if moderate changes occur simultaneously in both parameters (26, 27). For this reason, our group recently evaluated the optimal cut-off points for NAC positivity, establishing that a bilateral decrease of ≥24.48% in volume 2–6 cm measured by acoustic rhinometry is the optimal cut-off point to discriminate between patients with AR and NAR (29).

Nitric oxide (NO) is a metabolite synthesized by the respiratory mucosa as a compensatory mechanism during eosinophilic inflammation. Unlike fractional exhaled NO (FeNO), the usefulness of fractional nasal NO (FnNO) is controversial. FeNO does not only measures the NO from the lower airways, but from the entire respiratory mucosa. In this regard, the paranasal sinus mucosa is the main NO source of the airways due to the preferential expression of NO-synthase (30, 31). Nevertheless, the relative contribution of naso-sinusal NO to FeNO measurement remains to be established. On the other hand, when there is a high degree of edema, the ostia meatales are blocked and NO cannot be transported out of the sinuses. In this scenario, FnNO cannot reflect the extent of naso-sinusal (32) inflammation. Of note this phenomenon frequently occurs in patients with CRSwNP and with distinct rhinitis phenotypes.

Although mucosa-derived NO is a surrogate marker of airway eosinophilic, FeNO or FnNO measurements cannot establish the allergic etiology of rhinitis, so this technique is not useful for phenotyping patients with chronic nasal symptoms (Figure 2) (33, 34).

#### Olfactory Tests

Inflammatory diseases like chronic rhinitis or CRSwNP can produce olfactory dysfunctions by distinct mechanisms. On the one hand, in cases of complete nasal obstruction (e.g., CRSwNP) the odorants cannot reach the olfactory epithelium which is located distal to the upper turbinate (35). On the other hand, inflammatory phenomena can damage the neuroepithelium, thus inducing an irreversible loss of smell. Of note, there is no correlation between the severity of inflammation and the extent of damage in the olfactory epithelium.

More than 20% of AR patients have smell disturbances (36, 37). Different olfactory tests have been developed for clinical use, such as the Sniffin test (38), the University of Pennsylvania Smell Identification Test (UPSIT) (39) and the Barcelona Smell Test-24 (BAST-24) (40). The later test is composed of 24 odor detection, forced choice and odor identification scores; 20 of which allow the evaluation of the first cranial nerve and 4 of the fifth cranial nerve. The evaluation is performed by means of a questionnaire after being exposed for 5 s to each smell.

In conclusion, olfactory tests can be a useful tool to evaluate the smell disturbance in nasal pathologies such as CRS and AR (Figure 2) (40).

### *In vitro* Tests

In addition to clinical history and *in vivo* tests, several *in vitro* tests are also available to confirm the diagnosis of allergic phenotypes of rhinitis. These tests focus on the demonstration of sIgE (Figure 2) (41, 42).

#### Determination of Allergen-Specific IgE in Serum

Different *in vitro* tests are able to quantify sIgE (2). It is possible to quantify sIgE against the whole allergenic source or against molecular allergens. These methods to measure sIgE can evaluate only one allergen (singleplex assays) or several allergens simultaneously (multiplex assays). The first group includes the ImmunoCAP system (Thermo Fisher Scientific, TFS, Uppsala), which is the most widely used test; and the second group, the Immuno-Solid Phase Allergen Chip (ImmunoCAP ISAC from TFS), and the Allergy Explorer-ALEX system (Macroarray DX Wien, Austria) (43). It is also possible to customize multiplex allergen profiles through Euroimmun (Euroassay and Euroline) (44).

Both single and multiplex approaches have demonstrated high specificity and sensitivity (5, 44), being remarkable methods for identifying a sensitization state of patients of any age, independently of drug treatment. Nevertheless, SPT is still preferred for the identification of atopy due to its higher sensitivity and lower prize as compared to serum sIgE. However, the serum sIgE can be most convenient in some cases, especially in polysensitized and pediatric population (44–46), as numerous determinations can be performed using a small sample volume (41). Besides, with multiplex assays also permits a component-resolved-characterization of the atopic status, evaluating a wide set of aeroallergens, and to identify cross-reactive sensitization. This will also lead to the characterization of sensitization profile which will help in the prescription of a successful allergen immunotherapy.

The level of serum sIgE in AR patients usually correlates with *in vivo* test results. In fact, clinical history, SPT and serum sIgE represent the standard work-up for AR diagnosis and for deciding on allergen immunotherapy prescription in atopic patients (41). However, it cannot be useful to diagnose LAR or DAR. A recent study comparing sIgE levels against eight molecular allergens in grass pollen allergic individuals showed a good correlation between ISAC and ImmunoCAP results; however, ISAC showed higher values at higher IgE levels, while at low levels it missed slightly more samples, indicating sample dependency (47). Recently, comparisons of Euroline and ImmunoCAP results in both children and adults with seasonal AR showed a high correlation; nevertheless, the Euroline test appeared to overestimate serum specific IgE levels (48).

#### Basophil Activation Test

The basophil activation test (BAT) is a useful tool for the diagnosis of allergic phenotypes of rhinitis, as the presence of IgE-dependent allergen-specific responses in allergic patients can be demonstrated indirectly through a positive response in this patient-friendly method (4, 5, 49, 50). Using peripheral blood, it allows replicate *in vitro* type I hypersensitivity reactions suffered *in vivo* in patients after allergen exposure (51), being possible to analyze different allergens at the same time and without wash-out for antiallergic medication, in contrast to NAC. BAT has shown promising results for clinical diagnosis with a specificity >90% and a sensitivity of 50–66% for different allergens (5, 51, 52); however, its sensitivity is lower compared to SPT and it can trigger false-negative results (53).

The molecular pattern of sensitization can be disentangled with BAT, and may support AIT prescription in patients with LAR and DAR (5). The main advantage of BAT is the possibility to evaluate LAR patients, where undetectable levels of sIgE in serum and negative SPT can lead to misdiagnosis by confusing LAR and NAR. For them, BAT can be an essential tool because of its sensitivity for diagnosing IgE-mediated allergy (51). Of note, the IgE-dependent activation of basophils has been confirmed through BAT with wortmanin experiments (50, 54). Moreover, BAT does not require a previous NAC to improve its sensitivity (52, 54). Nevertheless, further studies are still required to confirm its diagnostic performance and to evaluate its cost-effectiveness (5, 52, 54).

#### Inflammatory Mediators at Local Level. Cytology and Nasal Lavage

In AR patients an alteration in the nasal mucosa due to inflammation exists, although these changes and its correlation with inflammatory cells and mediators is not well-characterized yet (55). Patients with allergic phenotypes of rhinitis show a T2 mucosal inflammatory pattern with an infiltrate of eosinophils, mast cells and T cells, leading to a nasal production of mediators such as tryptase and eosinophilic cationic protein (ECP) (41, 56), and sIgE (57). The analysis of nasal mucosal samples provides a lot of information, but it is an expensive technique and the clinical value of which is insufficiently evaluated (41).

There are both invasive and non-invasive techniques for sample collecting, although nasal lavage is the most used method, allowing the quantitative determination of cell distribution and inflammatory mediators (41, 55). Thus, mediators can be quantified in the supernatant of nasal lavage using different approaches, such as high-performance liquid chromatography and immunoassay (4, 57, 58), whereas nasal cytology can be analyzed in the sample pellet (55).

## Conclusions

In this review, we would like to emphasize the following concepts:

1. Allergic phenotypes of rhinitis are inflammatory disorders of the nasal mucosa affecting both atopic and non-atopic children and adults. These conditions are clinical manifestation of respiratory allergy, a prevalent, chronic and complex disease.
2. An etiologic allergological diagnosis, identifying the allergen/s which are clinically relevant for the patient is essential for a personalized treatment with specific AIT.
3. A specialized allergy diagnosis implemented by clinical history and allergy tests (NAC and BAT) should be considered when discrepancies exist between the pattern of nasal symptoms and atopy sensitization tests. This approach should be also followed in case of treatment failure.
4. Allergy and atopy are not equivalent phenomena or synonymous terms and basing allergy diagnosis on the sole determination of serum sIgE is associated with high rates of misdiagnosis.

## Author Contributions

CM conceptualized the article. AT-M, RJ, MS, and IE-G critically reviewed the literature and drafted the manuscript. AT-M and IE-G reviewed the manuscript and supervised the work of the other authors. All authors contributed to the article and approved the submitted version.

## Funding

This study was funded by grants from the Institute of Health Carlos III (ISCIII) of the Ministry of Economy and Competitiveness: PI18/00288 and PI20/01715. RETICS ARADyAL (RD16/0006/0001), research contracts Juan Rodes for IE-G (JR19/00029), Rio Hortega for AT-M (CM20/00160), and pFIS for RJ (F18/00133). Andalusian Regional Ministry of Health (PE-0039-2018) and Nicolas Monardes Program (RC-0004-2021). Grants were co-funded by the European Regional Development Fund (ERDF). Una manera de hacer Europa Andalucía se mueve con Europa.

## Conflict of Interest

The authors declare that the research was conducted in the absence of any commercial or financial relationships that could be construed as a potential conflict of interest.

## Publisher's Note

All claims expressed in this article are solely those of the authors and do not necessarily represent those of their affiliated organizations, or those of the publisher, the editors and the reviewers. Any product that may be evaluated in this article, or claim that may be made by its manufacturer, is not guaranteed or endorsed by the publisher.
